# The Immunity to Tuberculosis Possessed by Healthy Men

**Published:** 1903-01-17

**Authors:** 


### THE IMMUNITY TO TUBERCULOSIS POSSESSED BY HEALTHY MEN.

It is always well to see both sides of a question, hence we welcome the protest made by Sir Hugh R. Beevor[1] against the too ready acceptance of such extreme forms of the infection theory in regard to the causation of tuberculosis as are held by some. For the production of phthisis an infection agent is clearly necessary. But this simple statement by no means satisfies the public who straightaway label consumption an infectious disease, and proceed to treat it as such according to their lights. In so doing they have been well supported by many medical men, much it is to be feared to the detriment of a large class of sufferers. As showing how the fear of infection is at the present time working in the public mind, Sir Hugh Beevor tells us that in some parts of France they have had to abandon the idea of sending into the villages single patients of the working class, however strictly selected and although free from bacilli, and this for their cattle's sake rather than for their own health. Again, he says that the Bethel (Sullivan County) Board of Health has adopted an ordinance imposing a penalty of 10 dollars for the first offence and 50 dollars each week for entertaining a stranger or guest affected by tuberculosis other than immediate relatives or those depending on the family, and he adds that even if we had sanatoriums in plenty, and funds to replace the earnings of the bread-winners, it would still not be necessarily right to follow the lead of the peasants of France or the Board of Health of Bethel. We have to consider the proportionate importance of the infection which gives rise to the disease in those susceptible to it and the immunity which protects the mass of mankind from its attacks. As to the importance of this immunity it is pointed out that "from 1865 to 1897 the mortality from phthisis in boys from 10 to 15 years of age fell from 100 to 32. During these 32 years no action was taken owing to any idea of infection; indeed aggregation was increased by density of population and by school attendance to a three-fold extent, and risks of infection must have increased accordingly. Their diminished death-rate was due to avoidance of malnutrition, of the infection of fevers, and to a less degree to avoidance of bad air and faulty sanitation." It is evident that the defensive powers of a healthy man can cope with the attacks of the bacillus; and these powers are infinitely greater in him with regard to tubercle than in regard to the bacilli of other infectious diseases. "In tubercle, therefore, the first aim should be to reinforce such defence by attention to nutrition, ventilation and sanitation." Bearing these facts in mind Sir Hugh Beevor hopes that the medical profession will not encourage the public to avoid their tuberculous fellow-creatures. Such a course will not, in his opinion, diminish the amount of tubercle. It will, however, infallibly swell the ranks of the unemployed, it will depress the phthisical wage-earner's spirit, it will empty his pockets, and ultimately, by want and distress, reduce his family to that condition of low vitality which tubercle requires for a successful invasion. I would urge you, he says, as the educators of the public in these matters, distinctly to let it be known that tubercle is not highly infectious. "State that it is not a disease that requires isolation, and that only under certain quite exceptional conditions does it appear to be infectious at all. Insist that healthy people enjoy extraordinary immunity, that fresh air and open windows are the great armour against its attacks, and that hygiene will prove more valuable eventually than the isolation of the unwilling artisan or the excommunicatien of the clerk."

[1] Lancet, Jan. 10.

